# Miscellaneous

**Published:** 1892-05

**Authors:** 


### MISCELLANEOUS.

### THE STORY OF THE COAL.

In the library sat little Ruth, gazing earnestly at the bright glowing fire in the open grate, looking for the queer and beautiful pictures which I am sure all of you have seen some time or other in a coal fire. First there was the head of a dog, a splendid big dog, just like Watch, who lived next door, and was the terror of all tramps who dared set foot in the yard; there, over in the corner, were a horse and rider; presently the rider fell off and rolled headlong down into the glowing bed of coals. There was a castle, and Ruth, who had been reading of the castled Rhine, thought she could trace the river running in a narrow stream of flame across the grate.

"I wonder where coal comes from and what it is made of," she said aloud.

As she spoke a little red coal danced out and up to her with the evident intention of finding a resting place on her knee. As she started back in fear of being burned, a queer thin little voice said:

"Don't be afraid, little girl; I heard you wondering just now where the coal came from, and how it is made, and feeling in a very social, confidential mood, I thought I'd just come out and tell you all about it."

"Yes?" said Ruth, pulling her hair to see whether she was awake or not. "Yes, I would like to know, but I never knew before that coal could talk."

"Oh!" laughed the coal, and burned redder than ever. "Oh, yes, we can talk! I learned ages ago when the world was young."

"Why, how old are you?" queried Ruth. "I am only five, and I'm lots bigger than you."

"Well, I don't exactly know myself, but by the nearest computation I must be somewhere in the neighborhood of a few million years."

Ruth wondered very much what "computation" could be, that made any thing so dreadfully old, and said :

"Why, that's older than Methuselah !"

"Oh, bless you, yes !" answered the coal. "I lived ages before Methuselah was born."

"Where did you live ?" asked Ruth, now thoroughly interested in this antediluvian antiquity.

"Well, I lived down, deep down, deep down in the earth ; once, so long ago that I can scarcely remember it, I lived in a forest. I wasn't coal then, but grew on the bark of a fern ; millions of us spores, as you would call us in these days, fell to the ground, and with other vegetable growth were subjected to great heat, moisture and pressure. This was what is known by scholars as the carboniferous age."

"What big words it uses for such a little thing !" thought Ruth.

"For thousands of years, so many, I never could tell you how many, we lay there until one day we were broken off in pieces by men with picks, and found ourselves in a large damp place, lit by little lamps which the men wore in front of their caps. This was a coal mine, and the men were miners. We were next put in large cages, as they were called, and pulled up by machinery to the top of the mine. And then once more, after so many years, we saw the sunshine. After going through the process of screening, which is the separation of the larger from the smaller coal, we were loaded on cars and brought by railroad to this city, where we became first the property of the coal dealers and then of your father. And now what next will happen us I am sure I cannot tell ; we shall be thrown out to lie and shiver on an ash heap, I expect."

"I will keep you myself, always," said Ruth, "for telling me such a nice story. I will put you away in a pretty little box, as mamma does her rings, and that can be your house as long as I live."

"Much obliged, I'm sure," said the coal. "Speaking of your mother's rings, perhaps you do not know that I am second or third cousin to the diamond that flashes so brilliantly in one of them."

"You?" rather doubtfully exclaimed Ruth, not being able to see the slightest resemblance between this insignificant little lump of half-burned coal and the beautiful diamond which sparkled with all the colors of the rainbow on her mamma's hand.

"Yes, I. You see, the diamond is the purest form of carbon found. Now, I am not so closely related as the coal which is burned in your kitchen range ; that belongs to the family of Anthracite, which is much harder and contains more carbon than the Cannel family, of which I am a member. So you see while your kitchen coal may be first cousin, I come along third or fourth, but near enough to claim relationship."

"Ruth !" called mamma from the next room, "come, it is your bed time."

Ruth very thoughtfully went upstairs and made ready for bed, and holding closely in her chubby hand the entertaining coal, drifted away into the beautiful land of Nod.

### GRIZZLIES FIGHT TO A FINISH.

The following story of a combat between two enormous grizzlies is related by a hunter who witnessed it from the safe retreat of a tall tree, where he had fled from the approach of one of the brutes. He says that it lasted long enough for him to get over his scare and pay close attention to what was going on. He had been hunting, having started at daylight, and at 2 o'clock he was surprised by meeting a huge grizzly in the path. He did not have time to do any thing but climb a tree, and the old brute immediately laid siege and attempted to reach up at him. While this was going on he heard a noise up the trail, and there was another bear coming down towards his tree. He thought he was in for a night of it as the two would keep him there until he was starved or some one came to his assistance.

He was mistaken, however, for the two bears were evidently not friendly, and one of them undoubtedly thought that the other was poaching on his preserves, for the animals no sooner saw each other than they roared and rushed at each other, bent on fight.

Then ensued one of the wildest scenes ever witnessed in the mountains, a battle between the kings of the forest. The animals fought with a skill that showed them both to be the victor of many a previous conflict. They skirmished for position and boxed with the dexterity of trained pugilists. Suddenly one made a savage rush at the other and they were locked in an embrace that was terrific. They roared and howled and bit and clawed each other in a most horrible manner. The whole place was torn up, small trees being uprooted in the struggle of the enraged brutes. By an awful snort one tore away from the other, and then they began their sparring tactics again, but it was evident that the first bear had got much the worst of the tackle.

Round and round they went again until a second rush was made, and then came the most fearful death struggle. It was evident that one or the other would be dead before the other would give up the fight.

They fought savagely, biting and gouging, until one of the brutes fell, and the other got a grip on his throat, which soon ended the fight and the fallen bear's life. After biting and clawing the fallen enemy until there was no possibility of there being any life left, the victor set to work to eat his fallen foe.

---

### PURE FOOD.

The names of the gentlemen composing the Exposition Committee of the Food Manufacturers' Association, to be held at Madison Square Garden next October, are a guarantee to the public that this enterprise is only that which it is claimed to be—an earnest effort upon the part of honest men to improve the quality of our food supply.

The subject of adulterated food and medicines has been too long overlooked in this country, while every civilized nation under the sun has stringent laws to prevent adulteration in these things, except the United States.

The Paddock bill, now before Congress, is a simple one, perfectly constitutional; it passed the United States Senate without a roll call and should become a law.

### PACKER'S TAR SOAP.

Does just exactly what is claimed for it, quickly and thoroughly. The first application is attended with good results. By its use a bath is made a comfort instead of a torture. We have an excellent reason for saying this, as we have used a great variety of preparations for relieving irritations produced by hot weather and the confinement resulting from sickness, but the celerity with which the soap acted surprised us. The relief was immediate !

Ammonia and Borax.—The uses of ammonia and borax are manifold, and their value as household agents can scarcely be overrated. In the laundry, bath and kitchen, they are growing positively indispensable to the progressive housekeeper. The first is a valuable cleanser and disinfectant at the same time. By those who wish to do their work in the quickest and most effectual way, these remarkable facilitating agents are employed. As toilet articles they have no superior. A little ammonia in the bath will keep the skin healthy, deliciously sweet and clean, firm and fresh looking. It is found in many shampoo mixtures and is a thorough cleanser of the hair and scalp. Borax water is also excellent for washing the hair. Many prefer it for the face, as it renders the water very soft, and leaves the cuticle smooth as velvet. Weak ammonia water will clean hair brushes very rapidly and keep the bristles white and stiff. Weak borax water is a good dentifrice.

Fires in Bedrooms.—Gas stoves should never be used in sleeping rooms; they give a stuffy close feeling which is most unpleasant; while an open fireplace encourages ventilation, and thus makes the air pure and fresh, and is most agreeable. Children's sleeping rooms are better without fires at night, unless the weather is cold or very damp. They should be lighted early, not just as they are going to bed.

